# Dissecting the Genetic Susceptibility to Graves’ Disease in a Cohort of Patients of Italian Origin

**DOI:** 10.3389/fendo.2016.00021

**Published:** 2016-03-08

**Authors:** Angela Lombardi, Francesca Menconi, David Greenberg, Erlinda Concepcion, Marenza Leo, Roberto Rocchi, Michele Marinó, Mehdi Keddache, Yaron Tomer

**Affiliations:** ^1^Division of Endocrinology, Icahn School of Medicine at Mount Sinai, New York, NY, USA; ^2^Endocrinology, University Hospital of Pisa, Pisa, Italy; ^3^Battelle Center for Mathematical Medicine, Nationwide Children’s Hospital, Columbus, OH, USA; ^4^Cincinnati Children’s Hospital Medical Center, Cincinnati, OH, USA; ^5^Bronx VA Medical Center, Bronx, NY, USA

**Keywords:** Graves’ disease, thyroid diseases, genetic predisposition to disease, SNP association study, Italian patients

## Abstract

Graves’ disease (GD) is an autoimmune oligogenic disorder with a strong hereditary component. Several GD susceptibility genes have been identified and confirmed during the last two decades. However, there are very few studies that evaluated susceptibility genes for GD in specific geographic subsets. Previously, we mapped a new locus on chromosome 3q that was unique to GD families of Italian origin. In the present study, we used association analysis of single-nucleotide polymorphism (SNPs) at the 3q locus in a cohort of GD patients of Italian origin in order to prioritize the best candidates among the known genes in this locus to choose the one(s) best supported by the association. DNA samples were genotyped using the Illumina GoldenGate genotyping assay analyzing 690 SNP in the linked 3q locus covering all 124 linkage disequilibrium blocks in this locus. Candidate non-HLA (human-leukocyte-antigen) genes previously reported to be associated with GD and/or other autoimmune disorders were analyzed separately. Three SNPs in the 3q locus showed a nominal association (*p* < 0.05): rs13097181, rs763313, and rs6792646. Albeit these could not be further validated by multiple comparison correction, we were prioritizing candidate genes at a locus already known to harbor a GD-related gene, not hypothesis testing. Moreover, we found significant associations with the thyroid-stimulating hormone receptor (*TSHR*) gene, the cytotoxic T-lymphocyte antigen-4 (*CTLA-4*) gene, and the thyroglobulin (*TG*) gene. In conclusion, we identified three SNPs on chromosome 3q that may map a new GD susceptibility gene in this region which is unique to the Italian population. Furthermore, we confirmed that the *TSHR*, the *CTLA-4*, and the *TG* genes are associated with GD in Italians. Our findings highlight the influence of ethnicity and geographic variations on the genetic susceptibility to GD.

## Introduction

Graves’ disease (GD) is one of the most common autoimmune endocrine disorders characterized by goiter, hyperthyroidism and, in 10–25% of patients, ophthalmopathy (1, 2). GD is an antibody-mediated autoimmune disease, in which the pathogenic antibodies stimulate the thyroid-stimulating hormone (thyrotropin, TSH) receptor resulting in the clinical syndrome of goiter and hyperthyroidism. The pathogenesis of GD involves breakdown of central and peripheral tolerance, infiltration of the thyroid with thyroid-directed T-cells that escaped tolerance, and activation of B-cells to secrete TSH receptor (TSHR) stimulating antibodies (3).

While the exact etiology of GD has not been pinpointed yet, accumulating data, mainly obtained in twin studies, support a solid genetic predisposition to the disease, although the penetrance of the genetic determinants is about 22–35% (based on monozygotic twin concordance rates) (4–6). Several GD susceptibility genes have been identified and confirmed through linkage and association studies during the last three decades, including immune regulatory genes *HLA-DR*, (human leukocyte antigen – D related) cluster of differentiation 40 (*CD40*), *CTLA-4*, protein tyrosine phosphatase non-receptor type 22 (*PTPN22*), interleukin-2 receptor alpha chain (*CD25*), and thyroid-specific genes *TSHR* and *TG* (7). Moreover, several susceptibility genes for Graves’ ophthalmopathy have been proposed over the years based on small case–control association studies. These include *CTLA-4*, tumor necrosis factor-α (*TNF-*α), intercellular adhesion molecule-1 (*ICAM-1*), interferon-γ (*IFN-*γ) (8), insulin-like growth factor-1 receptor (*IGF-1R*) (9), suppressor of cytokine signaling 3 (*SOCS3*) (10), thyroid peroxidase (*TPO*) (11), calsequestrin 1 (*CASQ1*) (12), cysteine-rich angiogenic inducer 61 (*CYR61*), zinc finger protein 36 C3H type homog mouse (*ZFP36*), and stearoyl-coenzyme A desaturase (*SCD*) (13). However, none of the Graves’ ophthalmopathy specific genes have been confirmed.

Ethnicity and geographic variations influence the genetic susceptibility to GD. Previous studies demonstrated in Caucasian populations a positive and a negative association of GD with the serologically defined *HLA DR3* and *DR5* groups, respectively (14, 15). HLA-DRB1*0301 gave the highest positive predictive value for GD (15). *HLA* genes have been shown to be associated with GD in non-Caucasian populations as well, though the associated alleles were different from those observed in Caucasians (7, 16–18). For instance, the HLA-DRB1*0405/DQB1*0401 and the DRB3*020/DQA1*0501 haplotypes have been directly linked to GD in Asians (19) and in African-American (20) subjects, respectively. Notably, ethnic influences have been reported also for non-MHC genes; indeed linkage analysis in Caucasian families from different geographic regions showed different influences of the *CTLA-4* gene on GD development (21, 22), suggesting that even within the same ethnic group, geographic variations can have a strong effect on susceptibility to disease. Similarly, a recent study indicated that genetic polymorphisms at *CD25*, a gene that is associated with GD in Caucasians, do not exert a significant genetic effect on the development of GD in the Chinese Han population (23). Also, significant ethnic differences in the distribution of two germline TSHR polymorphisms (P52T and D727E) were found among the Chinese, Malays, and Indians (24).

Intriguingly, epidemiological surveys from various, mostly iodine sufficient, populations have shown a relatively comparable incidence of GD across Caucasian populations in different geographic regions (approximately 20–25/100,000/year). The comparable prevalence and incidence of GD in geographically distinct Caucasian populations may indicate that these populations share at least some of their susceptibility genes for GD, albeit unique GD genes for different populations must exist too (25). Nevertheless, there are very few studies that analyzed susceptibility genes for GD in specific geographic subsets of Caucasian GD patients. We have previously performed linkage analyses in a large dataset of multiplex GD families. Within our large dataset of GD families, an Italian subset of GD families did not show linkage to the *CD40* gene/locus on chromosome 20q in contrast to all other Caucasian populations we studied. Moreover, we mapped a new locus on chromosome 3q that was unique to the Italian subset of our GD families (26). These data suggest that there are unique susceptibility gene/loci within Caucasian GD patients, as determined by our subset of Italian GD families, and that distinct genes predispose to GD in different subsets of patients.

Our previous results demonstrate the importance of identifying subsets of patients within large datasets in order to map genes that may be subset-specific. Therefore, the goal of the present study was to fine map the 3q locus and evaluate known GD susceptibility genes in a cohort of GD patients of Italian origin.

## Materials and Methods

### Study Subjects

The project was approved by the Mount Sinai School of Medicine Institutional Review Board. We analyzed Caucasian GD patients of Italian origin. GD was diagnosed by (1) documented clinical and biochemical hyperthyroidism requiring treatment and (2) presence of TSH receptor antibodies and/or diffusely increase ^131^I uptake in the thyroid gland.

### Genotyping

DNA was extracted from whole blood using the Puregene kit (Gentra Systems, Minneapolis, MN, USA). DNA samples were genotyped using the Illumina GoldenGate genotyping assay as previously described (27). The GoldenGate single-nucleotide polymorphism (SNP) genotyping platform uses a discriminatory DNA polymerase and ligase to interrogate many SNPs simultaneously. The protocol is automated allowing high throughput and minimal errors. The GoldenGate genotyping assay utilized the BeadArray Reader. The GoldenGate assays were designed to analyze SNPs both in the linked locus on chromosome 3 and in a panel of GD candidate genes.

### Fine Mapping of the Chromosome 3q Locus

The chromosome 3q locus spans about 14 Mb and was fine mapped using a panel of 690 SNPs. Since the linkage analysis showed that there was a gene in the region, our goal in association mapping was to prioritize the best candidates among the known genes in this locus to choose the one(s) best supported by the association. Markers for fine mapping 3q were selected from the Illumina Infinium Human1M BeadChip panel leveraging the pre-selection by Illumina of markers focused on haplotype tagging and maximal allele frequency in the CEU population. The SNPs selected were sufficient to capture all alleles with an *r*^2^ > 0.8. The mean max *r*^2^ for our SNPs was 0.987. Therefore, our 690 SNPs gave more than sufficient coverage of the 3q locus.

Case–control association analyses for markers within the 3q locus were performed using the UNPHASED computer package.^1^ UNPHASED (28, 29) is a suite of programs for association analysis of multilocus haplotypes from unphased genotype data. We used the Cocaphase program (within the UNPHASED package) for case–control association analyses performing the χ^2^ test on all 690 SNPs simultaneously. The odds ratio (OR) was calculated by the method of Woolf (30).

### Linkage Disequilibrium Analysis

Linkage disequilibrium (LD) analysis was performed for the 3q locus using the Haploview program^2^ using SNP genotypes from the HapMap database (31). Haplotype analysis and haplotype association analysis of SNPs rs13097181, rs7633131, and 6792646 was performed with Haploview using our Italian GD and Italian controls genotypes.

### Candidate Gene Analysis

Non-*HLA* genes that have been previously reported to be associated with GD and/or other autoimmune disorders were analyzed separately using the χ^2^ test. These candidate genes included *TSHR*, *TG*, *CTLA-4*, *CD40*, interleukin 23 receptor (*IL23R*), *CD25*, *PTPN22*, forkhead box P3 (*FOXP3*), interferon regulatory factor 5 (*IRF5*), and toll-like receptor 4 (*TLR4*) genes (32–37).

### Power Calculations

Power calculations were performed using the CDC simulation software (Epi-Info, 7.1.3.3, CDC, Atlanta) (38). We assumed the population frequency of the susceptibility SNP allele to be ≥10% since we tested only common variants, and we used the gamete numbers since we performed allelic associations (39). The power calculations demonstrated that our dataset of 333 GD patients (666 chromosomes) and 117 controls (234 chromosomes) will give the study 80% power to detect a difference between the patients and the controls resulting in ORs of ≥2.0 with an alpha of 0.05. Therefore, our study was sufficiently powered to detect functionally significant differences between patients and controls.

## Results

### Characteristics of the Italian GD Population

We studied 333 Caucasian patients with GD of Italian origin. The average age at diagnosis was 40.0 ± 13.8 years (range 13–75 years), and there were 249 female and 84 male patients. The average age at ascertainment was 47.1 ± 13.7, and the average disease duration was 7.1 years. Additionally, 180 patients (54%) had goiter, 154 patients (46%) had Graves’ ophthalmopathy, and in 45 patients (14%), the ophthalmopathy status could not be confirmed. Control individuals of Italian origin (*n* = 117) had normal thyroid function, were negative for thyroid autoantibodies, and had no personal or family history of thyroid disease. There were 64 (55%) women and 53 men (45%). They were all over 18 years of age. However, the precise age at ascertainment was not available for all the controls.

### Fine Mapping of the Chromosome 3q Locus and Haplotype Analysis

**Figure 1:**
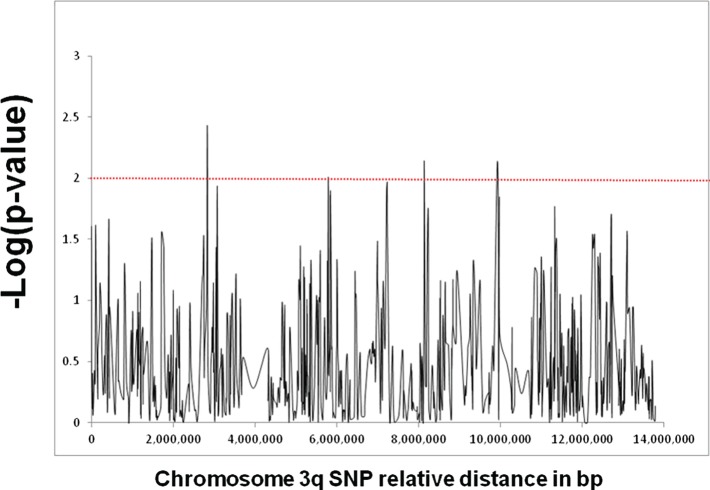
**Fine mapping analysis of the locus on chromosome 3q that showed strong linkage with GD in our subset of Italian GD families**. The locus spans approximately 14 Mb and was fine mapped using a panel of 690 informative SNPs. The *X*-axis shows the distance in base pair between the SNPs and the *Y*-axis shows the −log(*p*-value) as computed by the UNPHASED program. The red line corresponds to a *p*-value of 0.01. Three SNPs (rs13097181, rs7633131, and rs6792646) showed significant association with *p*-values <0.01.

Figure 1 shows the results of association analyses of the 690 SNPs used to fine map the chromosome 3q locus that is linked with GD in patients of Italian origin. Three SNPs showed a significant association: rs13097181 (*p* = 3.7 × 10^−3^, OR: 2.0), rs7633131 (*p* = 7.7 × 10^−3^, OR: 1.7), and rs6792646 (*p* = 7.8 × 10^−3^, OR: 1.5). LD analysis showed that the 3q locus contained 124 LD blocks. If we were testing the hypothesis that there is a GD gene in the region, it would have been necessary to correct for multiple testing which would make the associations not significant. However, we were not testing the hypothesis of a GD gene in this locus. Rather our goal was to prioritize candidate genes at a locus already known to harbor a GD-related gene, and the *p*-values were only used to prioritize.

Haplotype analysis performed using Haploview for SNPs rs13097181, rs7633131, and rs6792646 (using either the genotypes in our cohort or the HapMap genotypes) revealed that there was no LD between these three SNPs due to the large distances between them (5.3 Mb between rs13097181 and rs7633131; 1.8 Mb between rs7633131 and rs6792646). Therefore, no haplotypes were identified for these three SNPS, and no combination of alleles of these three SNPs was associated with GD.

### Candidate Gene Analysis

Several genes have been previously reported to be associated with GD in Caucasians and other ethnic groups (5, 40). We investigated a panel of genes previously shown to be linked with GD as well as genes associated with other autoimmune diseases (e.g., *IRF5* and *TLR4*, see Table 1). Among the candidate genes tested, we found associations with the *TSHR* gene (*p* = 3.94 × 10^−6^), the *CTLA-4* gene (*p* = 1 × 10^−3^), and the *TG* gene (*p* = 0.029).

**Table 1:** **Results of candidate gene analysis**.

Candidate gene	SNP	*p*-value
IL23R	rs2201841	0.622036
IL23R	rs11209026	0.259565
IL23R	rs10889677	0.786908
PTPN22	rs2476601	0.727479
**CTLA4**	**rs231775**	**0.00106852**
**CTLA4**	**rs3087243**	**0.0303233**
IRF5	rs4728142	0.45456
IRF5	rs10488631	0.769101
IRF5	rs12537284	0.56248
Tg	rs180194	0.384458
Tg	rs180223	0.624968
Tg	rs2069550	0.692976
Tg	rs853326	0.754905
**Tg**	**rs2069561**	**0.0292331**
Tg	rs2076740	0.182472
Tg	rs2294024	0.214108
TLR4	rs4986790	0.736433
TLR4	rs4986791	0.163881
CD25	rs706778	0.0630883
CD25	rs3118470	0.52791
CD25	rs41295061	0.498702
**TSHR**	**rs179247**	**3.94E−06**
**TSHR**	**rs3783948**	**0.00104205**
**TSHR**	**rs12101255**	**0.0356936**
CD40	rs1883832	0.464766
CD40	rs4810485	0.466903
FOXP3	rs6609857	0.743244
FOXP3	rs2294021	0.703636
FOXP3	rs2280883	0.747625
FOXP3	rs2232365	0.738291
FOXP3	rs3761549	0.496996

*Genes in bold are significantly associated with GD*.

## Discussion

A strong hereditary component in the pathogenesis of GD has long been recognized (41, 42). The inheritance of GD is complex, involving multiple genes with variable penetrance, and environmental and epigenetic factors also play a critical role in the etiology of the disease (5, 43, 44). Previous studies have shown differences in genetic associations with GD in different ethnic groups; however, most of these studies compared Caucasian patients to Asian patients.

In the present study, we dissected the genetic susceptibility in GD patients of Italian origin *via* fine mapping the Italian GD locus on chromosome 3q and analyzing several known susceptibility genes for GD. We identified three SNPs on chromosome 3q that were associated with GD in Italian patients, and we demonstrated that the *TSHR*, the *CTLA-4*, and the *TG* genes are associated with GD in Italians. Our results are consistent with previous findings suggesting the existence of unique autoimmunity susceptibility genes in Caucasian populations of different geographic regions (26, 45). Indeed, Marron et al. have shown considerable variability in the association of *CTLA-4* with type 1 diabetes among different Caucasian populations (46).

We tested 690 SNPs for association with GD at the 3q locus, a locus which a family study had previously been identified as containing a GD-related gene. Our goal in this study was not to identify a region containing disease-related gene but to prioritize which genes might be worth pursuing. Many of the 690 SNPs we typed shared LD blocks, meaning the association signals the SNPs produced were not independent of one another. The 3q locus that was fine mapped contained 124 LD blocks. Correcting for multiple testing using the Bonferroni correction for 124 tests would make the *p*-values for the three associated SNPs become not significant. However, since we were within a locus that we already determined to be linked to the disease, interpreting the Bonferroni-corrected *p*-values with the same intent as for a GWAS would miss our goal of determining which genes would be the best ones to examine in the next step of identifying the disease-related gene. Ideally, stronger association signals (*p*-values) would be better but the decision to investigate a specific patient population limited the sample size. Nonetheless, the results do give us important information that can be used to prioritize the known genes best supported by our association study.

The SNP within the chromosome 3q locus showing the strongest association with GD in Italians (rs13097181) maps to an intergenic region between the *EPHB1* gene (ephrin receptor B 1) and the *KY* gene (kyphoscoliosis peptidase). Notably, ephrin receptors make up the largest subgroup of the receptor tyrosine kinase family and are known to regulate T-cell activation, costimulation, and proliferation (47, 48), suggesting a potential functional role for *EPHB1* in the context of GD pathogenesis. The potential role, if any, of the *KY* gene, generally involved in processes of muscle growth (49), in GD pathophysiology remains to be determined. The other two chromosome 3q SNPs that were associated with GD in our population are rs7633131 and rs6792646. rs7633131 is an intronic SNP within the *CLSTN2* gene (calsyntenin 2), which does not affect the immune system or the thyroid (50). rs6792646 is an intergenic SNP located between the *GRK7* (G protein-coupled receptor kinase 7) gene and the *HMGN2P25* (high mobility group nucleosomal-binding domain 2 pseudogene 25) gene, two genes that do not seem to be functionally connected to GD (51, 52). Of course, the assumptions on potential mechanisms linking these genes to GD deserve further experimental validation. Nonetheless, disease-associated SNPs can also impact transcriptional mechanisms by modulating the binding of transcriptional factors or long-range interactions *via* genetic/epigenetic dysregulation (43, 53), eventually upregulating or downregulating gene transcription in order to generate a phenotype (54).

Genes that exert their effect early in the autoimmune response and control the breakdown in self-tolerance are strongly associated with GD. In the past two decades, several GD susceptibility genes have been identified and confirmed through linkage and association studies, including *HLA-DR*β*1-Arg74*, *CTLA-4*, *PTPN22*, *CD40*, *CD25*, *TG*, and the *TSHR* gene (55). *TG*, in particular, is an important candidate gene for GD, indeed the best model of human autoimmune thyroiditis is induced by immunizing mice with TG (56). Interestingly, GD patients have increased numbers of circulating T cells and B cells that proliferate in response to stimulation with TG (57). Recently, a significant positive correlation has been reported between serum TG levels and the presence and severity of ophthalmopathy in patients with GD. In these patients, TG levels also correlated to serum titers of TSHR antibodies, indicating that not only TSHR but also TG may be released from the thyroid gland in the course of a thyroiditis and home to the orbit where they become targets of autoantibodies and cytotoxic T lymphocytes (58, 59).

Furthermore, several genetic studies have demonstrated an association between thyroid autoimmunity and gene polymorphisms of proteins and enzymes associated with vitamin D functions (60–62). Indeed, in animal models, vitamin D administration efficiently prevented the induction of experimental autoimmune thyroiditis (63). Moreover, taking into account human studies, a high prevalence of vitamin D deficiency was reported both in patients with chronic autoimmune thyroiditis and in those with GD (64).

Consistent with previous reports (5, 40), among the candidate genes we tested, we found significant associations with the *TSHR*, *CTLA-4*, and *TG* genes. The *CTLA-4* gene had been previously implicated, in different ethnic groups, in diverse autoimmune disorders, including Addison’s disease (65), Hashimoto’s thyroiditis (66), Type 1 diabetes mellitus (22), multiple sclerosis (67), and GD (68). Of note, there was no significant association of GD with *CD40* in our dataset, which is in agreement with our previous finding that *CD40* was not linked with GD in a cohort of Italian families (69). Moreover, the TG SNP that is associated with GD in our Italian cohort is different than the one we previously identified in a cohort of North American Caucasian AITD patients, again underscoring the importance of ethnic variations in variants predisposing to GD.

The main limitation of our study is the relatively small size of our cohort. However, our cohort was ethnically homogenous, thereby increasing the power of our analysis.

In conclusion, we identified three SNPs on chromosome 3q that are associated with GD in Italian patients indicating potential GD susceptibility genes in this locus. Furthermore, we demonstrated that the *TSHR*, the *CTLA-4*, and the *TG* genes are associated with GD in Italians. Notably, there were no associations with *CD40*, *CD25*, and *FOXP3*. Our findings highlight the importance of ethnic variation in the association of different polymorphisms with GD even within the same candidate gene.

## Author Contributions

All authors have contributed significantly to the work, have read the manuscript, attest to the validity and legitimacy of the data and its interpretation, and agree to its submission.

## Conflict of Interest Statement

The authors declare that the research was conducted in the absence of any commercial or financial relationships that could be construed as a potential conflict of interest.

## Footnotes

^1^http://unphased.sourceforge.net/

^2^http://broad.mit.edu/mpg/haploview/
